# Coherence and Anticoherence Induced by Thermal Fields

**DOI:** 10.3390/e24050692

**Published:** 2022-05-13

**Authors:** Lihui Sun, Ya Liu, Chen Li, Kaikai Zhang, Wenxing Yang, Zbigniew Ficek

**Affiliations:** 1 Institute of Quantum Optics and Information Photonics, School of Physics and Optoelectronic Engineering, Yangtze University, Jingzhou 434023, China; lhsun@yangtzeu.edu.cn (L.S.); 2021710172@yangtzeu.edu.cn (Y.L.); 202006957@yangtzeu.edu.cn (C.L.); 202073044@yangtzeu.edu.cn (K.Z.); 2 Department of Physics, Southeast University, Nanjing 211189, China; 3 Quantum Optics and Engineering Division, Institute of Physics, University of Zielona Góra, Szafrana 4a, 65-516 Zielona Góra, Poland

**Keywords:** coherence, anticoherence, entanglement, nonlinear systems

## Abstract

Interesting coherence and correlations appear between superpositions of two bosonic modes when the modes are parametrically coupled to a third intermediate mode and are also coupled to external modes which are in thermal states of unequal mean photon numbers. Under such conditions, it is found that one of linear superpositions of the modes, which is effectively decoupled from the other modes, can be perfectly coherent with the other orthogonal superposition of the modes and can simultaneously exhibit anticoherence with the intermediate mode, which can give rise to entanglement between the modes. It is shown that the coherence effects have a substantial effect on the population distribution between the modes, which may result in lowering the population of the intermediate mode. This shows that the system can be employed to cool modes to lower temperatures. Furthermore, for appropriate thermal photon numbers and coupling strengths between the modes, it is found that entanglement between the directly coupled superposition and the intermediate modes may occur in a less restricted range of the number of the thermal photons such that the modes could be strongly entangled, even at large numbers of the thermal photons.

## 1. Introduction

The problem of the creation of coherence and correlations between quantum systems has attracted considerable interest over the years not only because of a basic desire to understand how coherence and correlations could be created but also because of their importance in determination of nonclassical states of quantum systems [1,2,3,4]. Various types of correlations can exist between quantum systems, and their importance in understanding properties of quantum systems is often discussed in connection with different phenomena. For example, interference and quantum beats are among the simplest examples of phenomena resulting from the presence of mutual coherence, the so-called first-order correlation between quantum systems. Nonclassical phenomena, such as squeezing and entanglement, result from the presence of a different kind of correlation, often referred to as anomalous correlations [5,6]. The mutual coherence resulting from the first-order correlation is produced by a constant or nearly constant phase difference between quantum systems [3,4,7,8]. There are, however, coherence effects resulting from higher-order correlations, e.g., the intensity correlations, which are possible even when the phase difference between systems is random [9,10,11,12].

Anomalous and intensity correlations are the natural products of a range of two-photon processes in which simultaneous or nearly simultaneous pairs of photons are produced [13,14]. Because each photon in the pair has no definite phase, there is no constant phase relation between them. Therefore, photons in the pair behave as mutually incoherent. This property has been observed experimentally in the process of parametric down conversion where pairs of photons, called the signal and idler photons, are produced [15,16]. Although the signal and idler photons are mutually incoherent, they are found in an entangled state which results from the anomalous correlation between the photons [17,18]. This observation suggests that the first-order correlation, which is responsible for the coherence and the anomalous correlation, are mutually exclusive. Following this observation, Mandel [19] proposed to call quantum systems exhibiting anomalous correlation as anticoherent.

The purpose of the present paper is to explore further possibilities to create coherence and anticoherence in a multipartite system. We consider a tripartite system composed of three coupled bosonic modes and investigate their coherence and anicoherence properties in an example of a three-mode optomechanical system, which consists of two cavity modes simultaneously coupled to the mode of a vibrating membrane. We assume that the cavity modes are affected by external input modes, which are in thermal states of unequal mean photon numbers. The difference in the mean number of photons of the input thermal fields constitutes an important and essential aspect of the work presented here. We will show how the populations of the modes and the correlations between them are sensitive to the population of the external thermal modes. When the external modes are in thermal states of different mean number of photons, we find that the steady-state populations of the modes can be dramatically altered, even to the point of the complete transfer of the population between the modes. Moreover, coherence and anticoherence, which may lead to entanglement between modes, can be established between modes which are completely decoupled from each other. This is certainly a surprising result since one would expect no correlations between decoupled modes affected by external thermal fields.

The paper is organized as follows. In Section 2, we introduce our model and the method of the evaluation of the dynamics of the systemś modes using an optomechanical system as an illustration. In Section 3, we study the properties of the steady-state population distribution between the modes. Section 4 is devoted to studying the correlations between the modes. We finish in Section 5 with the conclusion. In Appendix A, we present, as an illustration, a detailed derivation of the analytical expression for the steady-state population of the membrane mode.

## 2. Three-Mode System

The system we study consists of three parts; two modes whose fields are described by annihilation operators $a_{1}$ and $a_{2}$, coupled to a third mode whose field is described by an annihilation operator *b*. The modes $a_{1}$ and $a_{2}$ are coupled to mode *b* through the nonlinear (parametric) squeezing-type interactions. There is no direct coupling between modes $a_{1}$ and $a_{2}$. The Hamiltonian interaction for the three coupled modes is taken to be

(1) $$\begin{array}{c} H=\hbar g_{1}\left(a_{1}^{†}b^{†}+a_{1}b\right)+\hbar g_{2}\left(a_{2}^{†}b^{†}+a_{2}b\right), \end{array}$$

where $g_{1}$ and $g_{2}$ are the coupling constants between modes $a_{1}$ and *b*, and $a_{2}$ and *b*, respectively. The nonlinear squeezing-type interactions, as described by the Hamiltonian (1) can be created in a variety of systems. For example, squeezing-type interactions between several modes have been realized in linear optical schemes involving external source of squeezed light and networks of beamsplitters [20]. Another example where this type of interaction can be created is a ring cavity containing an atomic ensemble coupled to counter-propagating modes of the cavity [21,22].

**Figure 1:**
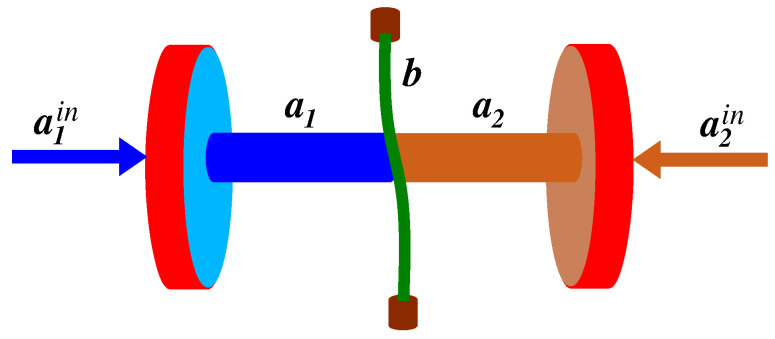
Schematic diagram of the system composed of two single-mode cavities sharing a vibrating membrane. The input fields to the cavities are in thermal states of unequal mean photon numbers.

A good example of such a system is an optomechanical system consisting of two single-mode cavities sharing an oscillating mirror [23], or a single-mode optical cavity coupled to two mechanical modes of a vibrating membrane [24,25,26]. The method of how to achieve the parametric-type interaction between cavity modes and mechanical (mirror or membrane) mode has been discussed in several review papers [27,28,29]. In what follows, we consider an optomechanical system similar to that considered by Paternostro et al. [23] where entanglement properties between the modes were studied, assuming that only the mirror mode is affected by external thermal fluctuations, i.e., the cavity modes were assumed to be in the ordinary vacuum states. This a a common practice in the study of the dynamics of optomechanical systems to assume that only the oscillating mirror or membrane is in contact with external modes (reservoir), being in a thermal state [30,31,32,33,34,35]. The ordinary vacuum states of the cavity modes are achieved by the coupling of the modes to an input (external) zero temperature modes. In practice, external modes are not in the ordinary vacuum but rather in non-zero temperature thermal states. Therefore, in what follows, we explore some correlation properties of a three-mode system, illustrated in Figure 1, assuming that the input modes to each of the cavities are in thermal states of unequal mean numbers of photons. The correlation properties of the modes affected by input thermal fields of unequal number of photons is the key point of the present work.

### 2.1. Time Evolution of the Modes

We start by writing a complete set of the quantum Langevin equations for the system which can be easily obtained from the Hamiltonian (1) when taking into account dissipation (damping) of the modes and coupling of the modes to external input modes. In the rotating frame, the equations are of the form

(2) $$\begin{array}{ccc} \dot{b} & = & -\gamma \;b+\frac{1}{2}i\left(g_{1}a_{1}^{†}+g_{2}a_{2}^{†}\right)+\sqrt{2\gamma }\;b^{in},\\ {\dot{a}}_{1} & = & -\kappa a_{1}+\frac{1}{2}ig_{1}b^{†}+\sqrt{2\kappa }\;a_{1}^{in},\\ {\dot{a}}_{2} & = & -\kappa a_{2}+\frac{1}{2}ig_{2}b^{†}+\sqrt{2\kappa }\;a_{2}^{in}, \end{array}$$

where γ is the decay rate of the membrane mode, and we have assumed the same decay rate κ for both cavity modes. Throughout Equation (3), operators $a_{1}^{in},a_{2}^{in}$ and $b^{in}$ are the input noise operators arising from the coupling of the modes to external modes (reservoirs). Here, we assume that the external modes are statistically independent, δ correlated, Gaussian, and in thermal states with

(3) $$\begin{array}{c} \begin{array}{ccc} \langle a_{i}^{in}\left(t\right)a_{i}^{in†}\left(t^{\prime }\right)\rangle & = & \left(n_{i}+1\right)\delta \left(t-t^{\prime }\right),\\ \langle a_{i}^{in†}\left(t\right)a_{i}^{in}\left(t^{\prime }\right)\rangle & = & n_{i}\delta \left(t-t^{\prime }\right),\\ \langle b^{in}\left(t\right)b^{in†}\left(t^{\prime }\right)\rangle & = & \left(n_{b}+1\right)\delta \left(t-t^{\prime }\right),\\ \langle b^{in†}\left(t\right)b^{in}\left(t^{\prime }\right)\rangle & = & n_{b}\delta \left(t-t^{\prime }\right), \end{array} \end{array}$$

where $n_{i}={\left(\exp\left\{\hbar \omega /k_{B}T_{i}\right\}-1\right)}^{-1}$ is the average number of photons in the external modes coupled to the *i*-th cavity mode of frequency ω and temperature $T_{i}$, and $n_{b}={\left(\exp\left\{\hbar \omega /k_{B}T_{b}\right\}-1\right)}^{-1}$ is the average number of photons in the external modes of temperature $T_{b}$ coupled to the membrane mode. Thus, in the absence of coupling to the membrane mode the cavity modes, $a_{1}$ and $a_{2}$ are in thermal states with mean numbers of photons $n_{1}$ and $n_{2}$, respectively, whereas the membrane is in thermal state with mean number of photons $n_{b}$.

### 2.2. Linear Superpositions of the Modes

It is seen from Equation (3) that mode *b* interacts simultaneously with both cavity modes. When a mode interacts simultaneously with two other modes, they may act collectively on the given mode. Therefore, it is more convenient to describe the dynamics of the system under consideration in terms of linear superpositions of the cavity modes. Thus, we can transform cavity annihilation operators to linear superpositions $a_{w}$ and $a_{u}$ of the form

(4) $$\begin{array}{c} \begin{array}{c} a_{w}=a_{1}\cos\theta +a_{2}\sin\theta ,\\ a_{u}=a_{1}\sin\theta -a_{2}\cos\theta , \end{array} \end{array}$$

and similarly, for the annihilation operators of the external input fields

(5) $$\begin{array}{c} \begin{array}{c} a_{w}^{in}=a_{1}^{in}\cos\theta +a_{2}^{in}\sin\theta ,\\ a_{u}^{in}=a_{1}^{in}\sin\theta -a_{2}^{in}\cos\theta , \end{array} \end{array}$$

where the mixing angle θ is given by $\tan\theta =g_{2}/g_{1}$. Hence in terms of the superposition modes, Equation (3) assumes the simplified form

(6) $$\begin{array}{ccc} \dot{b} & = & -\gamma b+\frac{1}{2}iga_{w}^{†}+\sqrt{2\gamma }\;b^{in},\\ {\dot{a}}_{w} & = & -\kappa a_{w}+\frac{1}{2}igb^{†}+\sqrt{2\kappa }\;a_{w}^{in},\\ {\dot{a}}_{u} & = & -\kappa a_{u}+\sqrt{2\kappa }\;a_{u}^{in}, \end{array}$$

where *g* is the effective coupling strength between the modes, $g=\sqrt{g_{1}^{2}+g_{2}^{2}}$.

For both analytical and numerical analyses, it is convenient to write the set of differential Equation (7) in a matrix form

(7) $$\begin{array}{c} \dot{v}=\mathrm{Av}+f_{in}, \end{array}$$

where $v^{T}=\left[b,a_{w}^{†},a_{u}\right],f_{in}^{T}=\left[\sqrt{2\gamma }b^{in},\sqrt{2\kappa }{\left(a_{w}^{in}\right)}^{†},\sqrt{2\kappa }a_{u}^{in}\right]$, and the drift matrix A is given by

(8) $$\begin{array}{c} A=\left(\begin{array}{ccc} -\gamma & \frac{1}{2}ig & 0\\ -\frac{1}{2}ig & -\kappa & 0\\ 0 & 0 & -\kappa \end{array}\right). \end{array}$$

**Figure 2:**
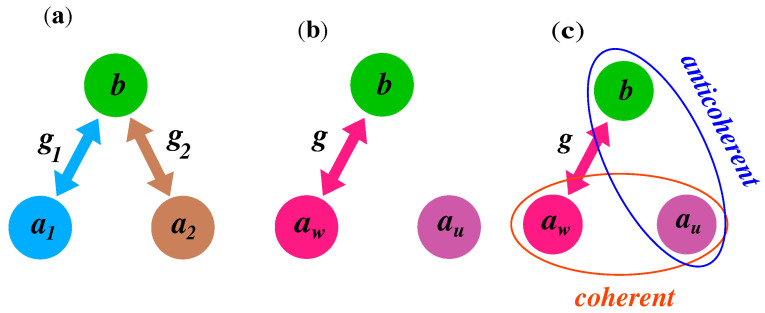
Coupling configurations between modes of the system. (**a**) Couplings between the mirror mode *b* and the cavity modes $a_{1}$ and $a_{2}$. (**b**) Couplings between the mode *b* and the superposition modes $a_{w}$ and $a_{u}$. (**c**) Illustration that the decoupled mode $a_{u}$ can be coherent with mode $a_{w}$ and anticoherent with mode *b*.

From Equation (4) we see that the superpositions of the modes can be controlled through θ by changing the relationship between coupling constants $g_{1}$ and $g_{2}$. However, the most important property seen from Equation (7) is that the superposition mode determined by the annihilation operator $a_{u}$ is effectively decoupled from modes $a_{w}$ and *b*. On the other hand, the mode $a_{w}$ is coupled to the membrane mode *b* with the effective coupling constant *g*. Despite the lack of the coupling of the $a_{u}$ mode to the remaining modes, we will show that the mode $a_{u}$ can exhibit first-order coherence with the mode $a_{w}$ and the so-called anticoherence with the mode *b*. The coupling configurations between different modes is shown in Figure 2.

Although the time-dependent solution of Equation (7) is complicated, see Appendix A, the steady-state solution is simple and easily obtained. Therefore, we will focus on the steady-state populations of the modes and correlations between them. We note that the solutions for the populations and correlation functions can be obtained from Equation (7) without approximations by a direct integration of the equations of motion. In the Appendix A, we present a detailed derivation of the steady-state population of the membrane mode.

## 3. Populations of the Modes

Let us first examine how different modes are populated in the presence of thermal fields of different mean photon numbers $n_{i}$. Solving Equation (7) for the steady-state, we find that the populations of the modes are

(9) $$\begin{array}{ccc} \langle b^{†}b\rangle & = & n_{b}+\frac{\kappa \left(n_{b}+1\right)g^{2}}{\left(\kappa +\gamma \right)\left(4\kappa \gamma -g^{2}\right)}+\frac{\kappa g^{2}}{\left(\kappa +\gamma \right)\left(4\kappa \gamma -g^{2}\right)}\left(n+\delta n\cos2\theta \right),\\ \langle a_{w}^{†}a_{w}\rangle & = & \frac{\gamma \left(n_{b}+1\right)g^{2}}{\left(\kappa +\gamma \right)\left(4\kappa \gamma -g^{2}\right)}+\left[1+\frac{\gamma g^{2}}{\left(\kappa +\gamma \right)\left(4\kappa \gamma -g^{2}\right)}\right]\left(n+\delta n\cos2\theta \right),\\ \langle a_{u}^{†}a_{u}\rangle & = & n-\delta n\cos2\theta , \end{array}$$

where $n=(n_{1}+n_{2})/2$ is the average number of photons, and $\delta n=(n_{1}-n_{2})/2$ is a difference between the average number of photons in the thermal fields coupled to the cavity modes. Note that δn can vary from −n to +n. The populations depend also on the coupling constant *g*, which cannot be arbitrarily large. The values of *g* are restricted to those at which the steady-state solutions for the populations are stable, i.e., are positive. It is easily seen from Equation (10) that the positivity of the populations requires $g<\sqrt{4\kappa \gamma }$. Alternatively, conditions for the stability of the steady-state solutions (10) can be determined by applying the Routh–Hurwitz criterion [36] to Equation (7), which says that the components of vector v decay to stable steady-state values when the determinant of the drift matrix A is negative. It is easily verified from Equation (8) that det (A)<0 when $g<\sqrt{4\kappa \gamma }$.

The first important fact we can derive from Equation (10) is that in the case of δn=0, the populations depend only on the effective coupling constant *g*. The difference δn≠0 induces a variation of the populations with the ratio of the coupling constants $g_{1}$ and $g_{2}$, determined by the mixing angle θ. This means that in the case of δn≠0, by changing the ratio $g_{2}/g_{1}$, i.e., by varying the mixing angle θ, one can change the population of the mode $a_{u}$ which is decoupled from the remaining modes $a_{w}$ and *b*. The transfer rate is proportional to δn, the difference of the thermal occupation of the modes $a_{1}$ and $a_{2}$. Thus, if only one of the cavity modes is subjected to thermal excitation and the other mode is in a vacuum state, then δn=±n, indicating that the thermal excitation of the cavity mode can be completely and reversibly transferred from modes *b* and $a_{w}$ to mode $a_{u}$.

**Figure 3:**
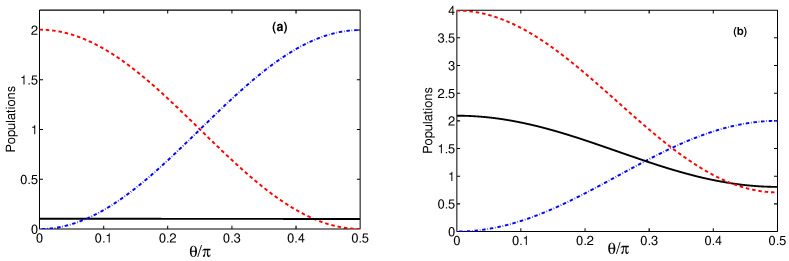
Populations of the modes plotted as a function of θ for γ=κ, n=1, δn=1, $n_{b}=0.1$ and two different values of the coupling strength *g*: (**a**) g=0.1κ, and (**b**) g=1.5κ. Black solid line shows $\langle b^{†}b\rangle$, dashed red line $\langle a_{w}^{†}a_{w}\rangle$, and dashed-dotted blue line $\langle a_{u}^{†}a_{u}\rangle$.

The results of our discussion of variations of the populations with θ when the difference δn≠0 are illustrated in Figure 3. We present here variations of the populations with θ for two different values of the effective coupling constant *g*. As it is seen, for a weak coupling g≪κ, the transfer of the population occurs between modes $a_{w}$ and $a_{u}$ only. The population of the mode *b* remains constant. Note the symmetry of the transfer process about θ=π/4 corresponding to $g_{1}=g_{2}$. For a strong coupling *g*, the transfer of the populations between the superposition modes is asymmetric about $\theta =\pi /4\;(g_{1}=g_{2})$ and is seen to be accompanied by a reduction of the population of mode *b*. In this case, the population is transferred to mode $a_{u}$ not only from mode $a_{w}$, but also from mode *b*. Lowering the population of the mode *b* implies that the system can be employed to cool the mode to a lower temperature. Thus, when δn≠0, it is possible to obtain dramatically reduced populations of the modes. In other words, keeping modes $a_{1}$ and $a_{2}$ at levels of different thermal occupations $\left(n_{1}\neq n_{2}\right)$ can work as a mechanism for the cooling of the membrane mode.

## 4. Correlations between the Modes

We now investigate the coherence and correlation effects between the modes when the modes are influenced by thermal fields. We assume that the thermal fields coupled to the cavity modes are of unequal numbers of thermal photons $n_{1}\neq n_{2}$, and the mirror mode is coupled a thermal state with the mean number of phonons $n_{b}$.

Different kinds of correlations can exist between the modes. Since the modes are in Gaussian states, which arises from the fact that the Hamiltonian (1) is quadratic, we consider only correlation functions up to a second order only. The correlation functions are expectation values of any combination of operators of two different modes. It is not difficult to show, using Equation (7), that in the steady state, there are the following non-zero correlation functions

(10) $$\begin{array}{ccc} \langle a_{u}^{†}a_{w}\rangle & = & \left[1+\frac{g^{2}}{8\kappa \left(\kappa +\gamma \right)-g^{2}}\right]\delta n\sin2\theta ,\\ \langle a_{w}b\rangle & = & \frac{2i\kappa \gamma g}{\left(\kappa +\gamma \right)\left(4\kappa \gamma -g^{2}\right)}\left(n+n_{b}+1+\delta n\cos2\theta \right),\\ \langle a_{u}b\rangle & = & \frac{4i\kappa g}{8\kappa \left(\kappa +\gamma \right)-g^{2}}\delta n\sin2\theta . \end{array}$$

and $\langle a_{w}^{†}b\rangle =\langle a_{u}^{†}b\rangle =\langle a_{w}a_{u}\rangle =0$. It is seen that the thermal fields of unequal photon numbers δn≠0 induce the first-order coherence between the superposition modes $a_{u}$ and $a_{w}$ determined by the function $\langle a_{u}^{†}a_{w}\rangle$, and a correlation between between $a_{u}$ and *b* modes determined by the function $\langle a_{u}b\rangle$, usually called an anomalous correlation function [5,6], or, after Mandel, called anticoherence [19]. As we already mentioned, the nonvanishing correlation function $\langle a_{u}^{†}a_{w}\rangle$ is the signature of the first-order coherence, which may lead to interference effects between the modes. It is well known that the nonvanishing anticoherence correlation functions $\langle a_{w}b\rangle$ and $\langle a_{u}b\rangle$ may lead to entanglement between the involved modes.

It is interesting that the mode $a_{u}$ which is decoupled from the other modes can exhibit first-order coherence with the mode $a_{w}$ and anticoherence with mode *b*. According to Equation (11), this can happen only when δn≠0. To demonstrate this, we examine in detail measures of the degree of coherence and anticoherence.

### 4.1. Degree of Coherence and Visibility

We already saw that the cross-correlation or mutual coherence function $\langle a_{u}^{†}a_{w}\rangle$ is different from zero when δn≠0. Therefore, the modes can be described as mutually coherent. The degree of coherence of the modes $a_{u}$ and $a_{w}$ is defined by the normalized quantity

(11) $$\begin{array}{c} \gamma_{uw}^{\left(1\right)}=\frac{\left|\langle a_{u}^{†}a_{w}\rangle \right|}{\sqrt{\langle a_{u}^{†}a_{u}\rangle \langle a_{w}^{†}a_{w}\rangle }}, \end{array}$$

whose values lie between 0 and 1.

**Figure 4:**
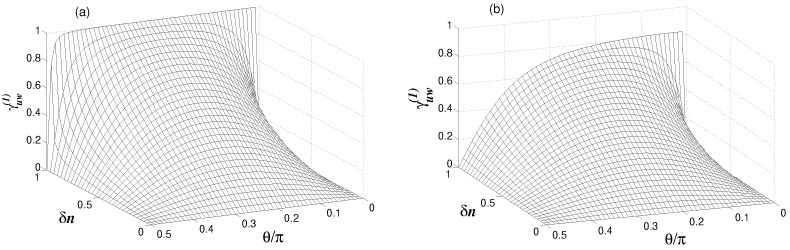
Variation of the degree of coherence between modes $a_{u}$ and $a_{w}$ with δn and θ for γ=κ, n=1, $n_{b}=0.1$ and two different values of the coupling strength *g*: (**a**) g=0.1κ, and (**b**) g=1.5κ.

In Figure 4, we plot the degree of coherence as a function of δn and θ. Notice that at δn=0, the modes are mutually incoherent, regardless of the value of θ. When δn≠0, the modes become mutually coherent. It is clearly seen that for a weak coupling between the modes g/κ≪1, illustrated in Figure 4a, the first-order coherence function is symmetric about θ=π/4, and becomes asymmetric when g/κ>1, the case corresponding to a strong coupling between modes, illustrated in Figure 4b. In this case, the degree of coherence is reduced in magnitude as θ increases. In the case of a weak coupling, an interesting situation is reached where the coherence attains its maximal value, i.e., the modes become mutually perfectly coherent when δn=n, i.e., when either $n_{1}$ or $n_{2}$ is equal to zero. On the other hand, in the strong coupling regime, the degree of coherence is always less than unity.

One can notice from Figure 4a that in the limit of δn=n, the modes are perfectly coherent when θ=0, but are completely incoherent when θ=π/2. The perfect coherence arises because the definite phase relationship between the modes $a_{1}$ and $a_{2}$ through the common coupling to the mode *b*.

Comparing the variation of $\gamma_{uw}^{\left(1\right)}$ with the variation of the populations of the modes, shown in Figure 3, we see that $\gamma_{uw}^{\left(1\right)}$ can be of equal unity regardless of the distribution of the population between the modes. This surprising behavior has been noticed before in systems of couple parametric downconverters [15,16,37,38,39,40], where interference effects were observed between the signal fields of the two downconverters with the degree of coherence $\gamma_{ij}^{\left(1\right)}=1$.

We saw that the modes can be perfectly mutually coherent regardless of the distribution of the population between them. However, the distribution of the population between the modes has an effect on the visibility of the interference pattern and distinguishability of the modes. The visibility V is determined by the coherence function

(12) $$\begin{array}{c} \left|V\right|=\frac{2|\langle a_{u}^{†}a_{w}\rangle |}{\langle a_{u}^{†}a_{u}\rangle +\langle a_{w}^{†}a_{w}\rangle }, \end{array}$$

whereas distinguishability is determined by the populations of the modes

(13) $$\begin{array}{c} \left|D\right|=\frac{|\langle a_{u}^{†}a_{u}\rangle -\langle a_{w}^{†}a_{w}\rangle |}{\langle a_{u}^{†}a_{u}\rangle +\langle a_{w}^{†}a_{w}\rangle }, \end{array}$$

The visibility and distinguishability obey the complementarity relation ${\left|V\right|}^{2}+{\left|D\right|}^{2}\leq 1$, in which the equality holds when the system is described by a pure state. When |D|=0, the modes are indistinguishable. On the other hand, when |D|=1, the modes are perfectly distinguished.

**Figure 5:**
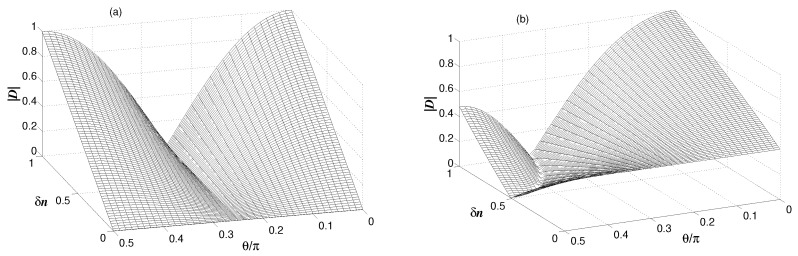
Dependence of the distinguishability |D| on δn and θ for γ=κ, n=1, $n_{b}=0.1$ and two different values of the coupling strength *g*: (**a**) g=0.1κ, and (**b**) g=1.5κ.

The distinguishability |D| is plotted in Figure 5 as a function of δn and θ. For δn=0, the distinguishability |D|=0 for all values of θ, indicating that in the case the cavity modes are affected by thermal fields of the same number of photons, and the superposition modes $a_{w}$ and $a_{u}$ are undistinguishable independent of the ratio $g_{2}/g_{1}$. For a weak coupling and δn≠0, illustrated in Figure 5a, the distinguishability varies between its minimal value |D|=0 at θ=π/4 to its maximal values at θ=0 and θ=π/2. In the completely asymmetric case where δn=±n, the distinguishability |D|=1. More precisely, the modes can be perfectly distinguishable (|D|=1) only if δn=n and either $g_{1}$ or $g_{2}$ is equal to zero. Thus, in the case of weak and equal coupling constants, θ=π/4, the modes are completely non-distinguishable, independent of δn. It is easy to understand if we refer to the fact that in the case of θ=π/4, the superpositions $a_{w}$ and $a_{u}$ are equally weighted, so that one can not predict from which mode a detected photon came from.

In the case of a strong coupling *g*, illustrated in Figure 5b, the distinguishability is strongly dependent on the relationship between $g_{1}$ and $g_{2}$. We see that the modes are always at least partly indistinguishable, except for δn=n and θ=0 at which |D|=1. Moreover, the modes are perfectly indistinguishable at θ≠π/4, i.e., when the modes are coupled to the membrane mode with unequal coupling strengths, $g_{1}\neq g_{2}$.

**Figure 6:**
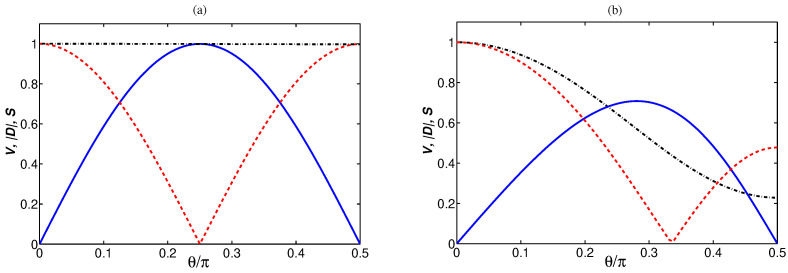
Close-up view of the variation of the distinguishability |D| (red dashed line) with θ at δn=n shown in Figure 5 together with the visibility |V| (blue solid line) and complementarity $S={\left|V\right|}^{2}+{\left|D\right|}^{2}$ (black dashed-dotted line) for γ=κ, n=1, $n_{b}=0.1$ and two different values of the coupling *g*: (**a**) g=0.1κ, and (**b**) g=1.5κ.

A close-up view of the variation of the distinguishability |D| with θ at δn=n is shown in Figure 6. We also plot the visibility |V| and the complementarity $S={\left|V\right|}^{2}+{\left|D\right|}^{2}$. The visibility vanishes only when $g_{1}=0$ or $g_{2}=0$, i.e., when one of the cavity modes is decoupled from the membrane mode. In the limit of a weak coupling, g≪κ, the visibility and distinguishability are perfectly mutually exclusive, and S=1 for all values of θ, indicating that independent of the ratio $g_{2}/g_{1}$, the system is in a pure state. On the other hand, in the limit of a strong coupling g>κ, they are no longer perfectly mutually exclusive, i.e., the visibility is greatest for $g_{1}\neq g_{2}$ and the maximum of the visibility does not correspond to the minimum of the distinguishability. Additionally, in this case, $V^{2}+{\left|D\right|}^{2}<1$, except θ=0 at which the modes are perfectly distinguishable. Thus, except θ=0, the system is in a mixed state. The mixed state results from the fact that in the strong coupling regime, not only the population from mode $a_{w}$, but also a population from the membrane mode *b* is transferred to mode $a_{u}$, as it is seen in Figure 3b.

### 4.2. Degree of Anticoherence and Entanglement

For the uncoupled modes $a_{u}$ and *b*, mutual coherence function $\langle a_{u}^{†}b\rangle$ is equal zero, and therefore the modes are mutually incoherent. Although the mutual coherence between the modes is equal to zero, it must not be thought that all correlations between the modes are zero. In fact, there are correlations present, but they are reflected by nonzero values of the correlation function $\langle a_{u}b\rangle$. This happens when δn≠0. Note that $\langle a_{u}b\rangle \neq 0$ is accompanied by $\langle a_{u}^{†}b\rangle =0$. Following Mandel [19], the correlation function $\langle a_{u}b\rangle$ is called the anticoherence function, and to quantify the degree of anticoherence, he introduced the measure of anticoherence

(14) $$\begin{array}{c} \gamma_{ub}^{\left(2\right)}=\frac{\left|\langle a_{u}b\rangle \right|}{\sqrt{\langle a_{u}^{†}b^{†}a_{u}b\rangle }}. \end{array}$$

The values of $\gamma_{ub}^{\left(2\right)}$ lie between 0 and 1.

When the modes obey the Gaussian statistics, then [41]

(15) $$\begin{array}{c} \langle a_{u}^{†}b^{†}a_{u}b\rangle =\langle a_{u}^{†}b^{†}\rangle \langle a_{u}b\rangle +\langle a_{u}^{†}b\rangle \langle b^{†}a_{u}\rangle +\langle a_{u}^{†}a_{u}\rangle \langle b^{†}b\rangle . \end{array}$$

Since $\langle a_{u}^{†}b\rangle =0$, Equation (14) then gives

(16) $$\begin{array}{c} \gamma_{ub}^{\left(2\right)}=\frac{\eta_{ub}}{\sqrt{\eta_{ub}^{2}+1}}, \end{array}$$

where

(17) $$\begin{array}{c} \eta_{ub}=\frac{\left|\langle a_{u}b\rangle \right|}{\sqrt{\langle a_{u}^{†}a_{u}\rangle \langle b^{†}b\rangle }}. \end{array}$$

is the normalized anomalous correlation function. Thus, the

The nonvanishing anticoherence corresponds to a situation in which the modes could be entangled. In order to connect anticoherence to entanglement, we consider the Cauchy–Schwarz inequality, which is often used to identify entanglement [3]. The Cauchy–Schwarz inequality for the modes $a_{u}$ and *b* is verified by reference to the so-called Cauchy–Schwartz parameter $\chi_{ub}$ involving the second-order correlation functions

(18) $$\begin{array}{c} \chi_{ub}=\frac{g_{u}^{\left(2\right)}g_{b}^{\left(2\right)}}{{\left(g_{ub}^{\left(2\right)}\right)}^{2}}, \end{array}$$

where

(19) $$\begin{array}{c} g_{ub}^{\left(2\right)}=\frac{\langle a_{u}^{†}b^{†}a_{u}b\rangle }{\langle a_{u}^{†}a_{u}\rangle \langle b^{†}b\rangle } \end{array}$$

is the normalized second-order cross correlation function, and

(20) $$\begin{array}{c} g_{u}^{\left(2\right)}=\frac{\langle a_{u}^{†2}a_{u}^{2}\rangle }{{\langle a_{u}^{†}a_{u}\rangle }^{2}},\;g_{b}^{\left(2\right)}=\frac{\langle b^{†2}b^{2}\rangle }{{\langle b^{†}b\rangle }^{2}}, \end{array}$$

are the normalized intensity autocorrelation functions of the modes $a_{u}$ and *b*, respectively.

Using the Gaussian-mode decomposition (15), the correlation functions can be readily related to the coherence functions

(21) $$\begin{array}{ccc} g_{i}^{\left(2\right)} & = & 2+\eta_{ii}^{2},\;i=u,b,\\ g_{ub}^{\left(2\right)} & = & 1+{\left(\gamma_{ub}^{\left(1\right)}\right)}^{2}+\eta_{ub}^{2}. \end{array}$$

Since in our case, $\eta_{uu}=\eta_{bb}=\gamma_{ub}^{\left(1\right)}=0$, the Cauchy–Schwarz parameter takes the form

(22) $$\begin{array}{c} \chi_{ub}=\frac{4}{{\left(1+\eta_{ub}^{2}\right)}^{2}}, \end{array}$$

which can be expressed in terms of the degree of the anticoherence as

(23) $$\begin{array}{c} \chi_{ub}=4{\left[1-{\left(\gamma_{ub}^{\left(2\right)}\right)}^{2}\right]}^{2}. \end{array}$$

To examine the occurrence of entanglement, we must check whether the Cauchy–Schwarz inequality $\left(\chi_{ub}>1\right)$ is violated. From Equation (23), we see that the condition that the modes are anticoherent, i.e., $\gamma_{ub}^{\left(2\right)}$ is a necessary but not sufficient condition for entanglement between the modes. In other words, the modes could be anticoherent but not enough to obtain $\chi_{ub}<1$. It is easily verified that for the Cauchy–Schwarz inequality to be violated, it is necessary that $\gamma_{ub}^{\left(2\right)}>1/\sqrt{2}$. Thus, for two modes to be entangled, they should be anticoherent to a degree about 71%.

**Figure 7:**
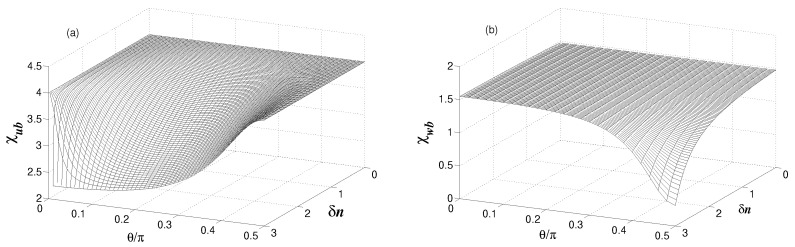
Variation of the Cauchy–Schwarz parameters (**a**) $\chi_{ub}$ and (**b**) $\chi_{wb}$ with δn and θ for γ=κ, n=3, $n_{b}=0.1$ and g=κ.

Figure 7a shows the Cauchy–Schwarz parameter $\chi_{ub}$ as a function of δn and θ. It is clearly seen that the parameter $\chi_{ub}$ is reduced below its maximal value $\chi_{ub}=4$ when δn≠0. The parameter $\chi_{ub}$ decreases to a minimum value at δn=n, but unfortunately the minimal value is not smaller than the threshold for entanglement $\left(\chi_{ub}=1\right)$. This indicates that the anticoherence between the modes is not strong enough for the modes $a_{u}$ and *b* to be entangled.

Although modes $a_{u}$ and *b* are not entangled, there could be entanglement between modes $a_{w}$ and *b*, which are directly coupled to each other. The results for the Cauchy–Schwarz parameter $\chi_{wb}$ are shown in Figure 7b. It is seen that for certain values of δn and θ, the parameter $\chi_{wb}$ can be reduced below the threshold for entanglement. It was noticed before that in the case when the cavity modes are affected by thermal fields of the same photon numbers $\left(n_{1}=n_{2}=n\right)$, entanglement between cavity mode and the membrane mode is restricted to very small values of n<1/2. The results shown in Figure 7b are in sharp contrast to the case of equal number of thermal excitations, where entanglement is restricted to very small values of *n* and indicate quite clearly that in the case of unequal photon numbers $\left(n_{1}\neq n_{2}\right)$, entanglement between the modes can be observed, even for large values of *n*.

In physical terms, we may attribute the appearance of entanglement between modes $a_{w}$ and *b* when $n_{1}\neq n_{2}$ to the fact that a part of the population of the modes, which has a destructive effect on entanglement, is transferred and stored in the decoupled mode $a_{u}$.

Before concluding, we note that although we have discussed and graphically illustrated the coherence and anticoherence properties of the modes only for the case of equal damping rates of the modes, γ=κ, analogous results are obtained in the experimentally realistic case of γ≪κ [27,28,29].

**Figure 8:**
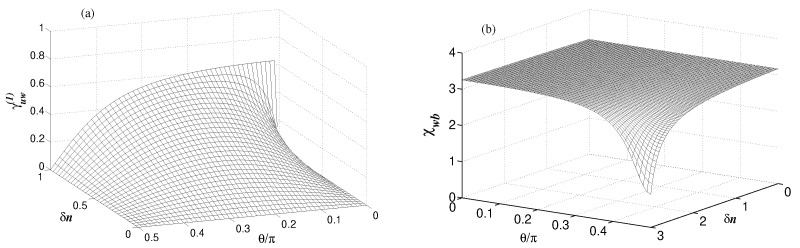
(**a**) Variation of the degree of coherence $\gamma_{uw}^{\left(1\right)}$ with δn and θ for γ=0.01κ, n=1, $n_{b}=0.1$ and g=0.19κ. (**b**) Variation of the Cauchy-Schwarz parameter $\chi_{wb}$ with δn and θ for γ=0.01κ, n=3, $n_{b}=0.1$ and g=0.19κ.

As an illustration, in Figure 8, we plot $\gamma_{uw}^{\left(1\right)}$ and $\chi_{wb}$ for γ=0.01κ. Comparing the results with those presented in Figure 4b and Figure 7b we saw that $\gamma_{uw}^{\left(1\right)}$ and $\chi_{wb}$ behave in qualitatively the same manner as for γ=κ. While the maximal value of the coherence $\gamma_{uw}^{\left(1\right)}$ between uncoupled modes is reduced for γ≪κ compared with Figure 4b, it is still nonzero over the entire range of δn≠0. Similarly, although the parameter $\chi_{wb}$ has risen for γ≪κ compared with Figure 7b, the region near δn=n still shows reduction of $\chi_{wb}$ below the threshold for entanglement.

## 5. Conclusions

We considered coherence properties between modes of a three-mode optomechanical system composed of two cavity modes simultaneously coupled to a membrane mode. We obtained analytical solutions for the steady-state populations of the modes and correlation functions describing coherence effects between the modes. Working in terms of linear superpositions of the cavity modes, we showed that one of the linear superpositions can be completely decoupled from the remaining modes. In spite of this, we found that the decoupled superposition can be completely coherent with the other superposition modes and can simultaneously exhibit anticoherence with the membrane mode. A detailed analysis showed that these correlation effects can happen only when the cavity modes are affected by the external input modes being in thermal states of unequal average photon numbers. In particular, we found that the coherences have a substantial effect on population distribution between the modes such that the population can be reversibly transferred between the superposition modes. The transfer of the population can lead to lowering of the population of the membrane mode. Therefore, the system can be considered as an alternative way to cool modes to lower temperatures. We also showed that a difference of the average numbers of photons in the thermal fields may affect entanglement between the superposition mode directly coupled to the membrane mode such that it may occur in a less restricted range of the number of thermal photons. In other words, the modes could be entangled, even with large numbers of thermal photons.

## Data Availability

Not applicable.

## Appendix A. Evaluation of the Steady-State Population of the Membrane Mode

In this Appendix, we provide some details of the derivation of the analytical expression for the steady-state population of the mode *b*. Using Equation (7), we find that a former integration of the equations for *b* and $a_{w}$ leads to

(A1) $$\begin{array}{c} b\left(t\right)=b\left(0\right)e^{-\gamma t}+\frac{1}{2}ige^{-\gamma t}\int_{0}^{t}dt^{\prime }a_{w}^{†}\left(t^{\prime }\right)e^{\gamma t^{\prime }}+\sqrt{2\gamma }e^{-\gamma t}\int_{0}^{t}dt^{\prime }b^{in}\left(t^{\prime }\right)e^{\gamma t^{\prime }}, \end{array}$$

(A2) $$\begin{array}{c} a_{w}\left(t\right)=a_{w}\left(0\right)e^{-\kappa t}+\frac{1}{2}ige^{-\kappa t}\int_{0}^{t}dt^{\prime }b^{†}\left(t^{\prime }\right)e^{\kappa t^{\prime }}+\sqrt{2\kappa }\;e^{-\kappa t}\int_{0}^{t}dt^{\prime }a_{w}^{in}\left(t^{\prime }\right)e^{\kappa t^{\prime }}. \end{array}$$

Substituting the expression for $a_{w}^{†}$ into Equation (A1) and using the double integration rule

(A3) $$\begin{array}{c} \int_{0}^{t}dt^{\prime }A\left(t^{\prime }\right)\int_{0}^{t}dt^{\prime \prime }B\left(t^{\prime \prime }\right)=\int_{0}^{t^{\prime }}dt^{\prime }B\left(t^{\prime }\right)\int_{t^{\prime }}^{t}dt^{\prime \prime }A\left(t^{\prime \prime }\right) \end{array}$$

we find that the expression for b(t) can be written as

(A4) $$\begin{array}{c} b\left(t\right)=y\left(t\right)+\int_{0}^{t}dt^{\prime }K\left(t,t^{\prime }\right)b\left(t^{\prime }\right), \end{array}$$

where $K(t,t^{\prime })$ is the kernel of the integral of the form

(A5) $$\begin{array}{c} K\left(t,t^{\prime }\right)=\frac{g^{2}}{4(\gamma -\kappa )}\left(e^{-\kappa (t-t^{\prime })}-e^{-\gamma (t-t^{\prime })}\right), \end{array}$$

and the term y(t) has the form

(A6) $$\begin{array}{ccc} y(t) & = & b\left(0\right)e^{-\gamma t}-\frac{ga_{w}^{†}\left(0\right)}{2(\gamma -\kappa )}\left[e^{-\kappa t}-e^{-\gamma t}\right]+\sqrt{2\gamma }\int_{0}^{t}dt^{\prime }b^{in†}\left(t^{\prime }\right)e^{-\gamma (t-t^{\prime })}\\ & + & i\frac{\sqrt{2\kappa }g}{2(\gamma -\kappa )}\int_{0}^{t}dt^{\prime }a_{w}^{in†}\left(t^{\prime }\right)\left[e^{-\kappa (t-t^{\prime })}-e^{-\gamma (t-t^{\prime })}\right]. \end{array}$$

It is seen that the kernel $K(t,t^{\prime })$ depends only on the time difference $t-t^{\prime }$, and may be written in the form

(A7) $$\begin{array}{c} K\left(t,t^{\prime }\right)=\frac{g^{2}}{4(\gamma -\kappa )}H\left(t,t^{\prime }\right)=\lambda H\left(t,t^{\prime }\right), \end{array}$$

where $\lambda =g^{2}/4\left(\gamma -\kappa \right)$.

The integral Equation (A4) can be solved using the Laplace transformation. Thus if

(A8) $$\begin{array}{c} \int_{0}^{t}dt^{\prime }H\left(t^{\prime }\right)e^{-pt^{\prime }}=H\left(p\right),\\ \;\int_{0}^{t}dt^{\prime }y\left(t^{\prime }\right)e^{-pt^{\prime }}=y\left(p\right),\\ \;\int_{0}^{t}dt^{\prime }b\left(t^{\prime }\right)e^{-pt^{\prime }}=b\left(p\right), \end{array}$$

we obtain from Equation (A4)

(A9) $$\begin{array}{c} b\left(p\right)=\frac{y(p)}{1-\lambda \;H(p)}, \end{array}$$

where

(A10) $$\begin{array}{c} H\left(p\right)=\frac{1}{p+\kappa }\;-\frac{1}{p+\gamma }, \end{array}$$

and y(p) is

(A11) $$\begin{array}{c} \begin{array}{ccc} y(p) & = & \left\{b\left(0\right)+\sqrt{2\gamma }B\left(p\right)+\frac{g}{2(\gamma -\kappa )}\left[a_{w}^{†}\left(0\right)-i\sqrt{2\kappa }A_{w}^{†}\left(p\right)\right]\right\}\frac{1}{p+\gamma }\\ & - & \frac{g}{2(\gamma -\kappa )}\left[a_{w}^{†}\left(0\right)+i\sqrt{2\kappa }A_{w}^{†}\left(p\right)\right]\frac{1}{p+\kappa }, \end{array} \end{array}$$

with

(A12) $$\begin{array}{ccc} B(p) & = & \int_{0}^{t}dt^{\prime }b^{in}\left(t^{\prime }\right)e^{-pt^{\prime }},\\ A^{†}\left(p\right) & = & \int_{0}^{t}dt^{\prime }a_{w}^{in†}\left(t^{\prime }\right)e^{-pt^{\prime }}. \end{array}$$

Substituting the solution (A10) for H(p) into Equation (A9), we readily find

(A13) $$\begin{array}{c} b\left(p\right)=\frac{y(p)(p+\kappa )(p+\gamma )}{\left(p+\kappa )(p+\gamma )-\lambda \;(\gamma -\kappa \right)}. \end{array}$$

Having available the Laplace transform b(p), we find b(t) simply by taking the inverse of the Laplace transformation (A13). We then obtain

(A14) $$\begin{array}{c} \begin{array}{ccc} b(t) & = & \sum_{i=1}^{2}\left(p-p_{i}\right)b\left(p_{i}\right)e^{p_{i}t}\\ & = & b\left(0\right)\left[\frac{\left(\kappa -\gamma \right)}{\Delta }\sinh\left(\frac{1}{2}\Delta t\right)+\cosh\left(\frac{1}{2}\Delta t\right)\right]e^{-\frac{1}{2}\left(\kappa +\gamma \right)t}+a_{w}^{†}\left(0\right)\frac{ig}{\Delta }\sinh\left(\frac{1}{2}\Delta t\right)e^{-\frac{1}{2}\left(\kappa +\gamma \right)t}\\ & + & \frac{ig\sqrt{2\kappa }}{2\Delta }\left[A^{†}\left(p_{1}\right)e^{\frac{1}{2}\Delta t}-A^{†}\left(p_{2}\right)e^{-\frac{1}{2}\Delta t}\right]e^{-\frac{1}{2}\left(\kappa +\gamma \right)t}\\ & + & \frac{\sqrt[{}]{2\gamma }}{2\Delta }\left\{\left[\left(\kappa -\gamma \right)+\Delta \right]B\left(p_{1}\right)e^{\frac{1}{2}\Delta t}-\left[\left(\kappa -\gamma \right)-\Delta \right]B\left(p_{2}\right)e^{-\frac{1}{2}\Delta t}\right\}e^{-\frac{1}{2}\left(\kappa +\gamma \right)t}, \end{array} \end{array}$$

where

(A15) $$\begin{array}{c} p_{1,2}=\frac{1}{2}\left(\kappa +\gamma \right)\pm \frac{1}{2}\sqrt[{}]{{\left(\kappa -\gamma \right)}^{2}+g^{2}} \end{array}$$

are roots of the quadratic equation

(A16) $$\begin{array}{c} \left(p+\kappa \right)\left(p+\gamma \right)-\frac{1}{4}g^{2}=0, \end{array}$$

and $\Delta =\sqrt{{\left(\kappa -\gamma \right)}^{2}+g^{2}}$.

We can use the solution b(t) to find the population of the mode *b* simply multiplying b(t) from the left by $b^{†}\left(t\right)$ and then taking the expectation value. We thus find

(A17) $$\begin{array}{c} \begin{array}{ccc} \langle b^{†}\left(t\right)b\left(t\right)\rangle & = & \langle b^{†}\left(0\right)b\left(0\right)\rangle {\left[\frac{\left(\kappa -\gamma \right)}{\Delta }\sinh\left(\frac{1}{2}\Delta t\right)+\cosh\left(\frac{1}{2}\Delta t\right)\right]}^{2}e^{-(\kappa +\gamma )t}\\ & + & \left[\langle a_{w}^{†}\left(0\right)a_{w}\left(0\right)\rangle +1\right]\frac{g^{2}}{\Delta^{2}}\sinh^{2}\left(\frac{1}{2}\Delta t\right)e^{-(\kappa +\gamma )t}\\ & + & \frac{\kappa g^{2}}{2\Delta^{2}}\left[\langle A\left(p_{1}\right)A^{†}\left(p_{1}\right)\rangle e^{\Delta t}+\langle A\left(p_{2}\right)A^{†}\left(p_{2}\right)\rangle e^{-\Delta t}\right.\\ & - & \left.\langle A\left(p_{1}\right)A^{†}\left(p_{2}\right)\rangle -\langle A\left(p_{2}\right)A^{†}\left(p_{1}\right)\rangle \right]e^{-(\kappa +\gamma )t}\\ & + & \frac{\gamma }{2\Delta^{2}}\left\{{\left[\left(\kappa -\gamma \right)+\Delta \right]}^{2}\langle B^{†}\left(p_{1}\right)B\left(p_{1}\right)\rangle e^{\Delta t}+{\left[\left(\kappa -\gamma \right)-\Delta \right]}^{2}\langle B^{†}\left(p_{2}\right)B\left(p_{2}\right)\rangle e^{-\Delta t}\right.\\ & + & \left.g^{2}\left(\langle B^{†}\left(p_{1}\right)B\left(p_{2}\right)\rangle +\langle B^{†}\left(p_{2}\right)B\left(p_{1}\right)\rangle \right)\right\}e^{-(\kappa +\gamma )t}, \end{array} \end{array}$$

where

(A18) $$\begin{array}{c} \langle A\left(p_{i}\right)A^{†}\left(p_{i}\right)\rangle =\int_{0}^{t}dt^{\prime }\int_{0}^{t}dt^{\prime \prime }\langle a_{w}^{in}\left(t^{\prime }\right)a_{w}^{in†}\left(t^{\prime \prime }\right)\rangle e^{-p_{i}\left(t^{\prime }+t^{\prime \prime }\right)},\;i=1,2, \end{array}$$

and

(A19) $$\begin{array}{c} \langle B^{†}\left(p_{i}\right)B\left(p_{i}\right)\rangle =\int_{0}^{t}dt^{\prime }\int_{0}^{t}dt^{\prime \prime }\langle b^{in†}\left(t^{\prime }\right)b^{in}\left(t^{\prime \prime }\right)\rangle e^{-p_{i}\left(t^{\prime }+t^{\prime \prime }\right)},\;i=1,2. \end{array}$$

Since $\langle b^{in†}\left(t^{\prime }\right)b^{in}\left(t^{\prime \prime }\right)\rangle =n_{b}\delta \left(t^{\prime }-t^{\prime \prime }\right)$, we get

(A20) $$\begin{array}{c} \langle B^{†}\left(p_{i}\right)B\left(p_{i}\right)\rangle =n_{b}\int_{0}^{t}dt^{\prime }e^{-2p_{i}t^{\prime }}=\frac{n_{b}}{2p_{i}}\left(1-e^{-2p_{i}t}\right),\;i=1,2, \end{array}$$

and

(A21) $$\begin{array}{c} \langle B^{†}\left(p_{1}\right)B\left(p_{2}\right)\rangle =\langle B^{†}\left(p_{2}\right)B\left(p_{1}\right)\rangle =\frac{n_{b}}{p_{1}+p_{2}}\left[1-e^{-(p_{1}+p_{2})t}\right]. \end{array}$$

Similarly, since

(A22) $$\begin{array}{c} \langle a_{w}^{in}\left(t^{\prime }\right)a_{w}^{in†}\left(t^{\prime \prime }\right)\rangle =\left(n_{w}+1\right)\delta \left(t^{\prime }-t^{\prime \prime }\right), \end{array}$$

where $n_{w}=\left(g_{1}^{2}n_{1}+g_{2}^{2}n_{2}\right)/g^{2}$, we get

(A23) $$\begin{array}{c} \langle A\left(p_{1}\right)A^{†}\left(p_{1}\right)\rangle =\left(n_{w}+1\right)\int_{0}^{t}dt^{\prime }e^{-2p_{1}t^{\prime }}=\frac{\left(n_{w}+1\right)}{(\kappa +\gamma )-\Delta }\left(e^{(\kappa +\gamma -\Delta )t}-1\right), \end{array}$$

and

(A24) $$\begin{array}{ccc} \langle A\left(p_{2}\right)A^{†}\left(p_{2}\right)\rangle & = & \frac{\left(n_{w}+1\right)}{(\kappa +\gamma )+\Delta }\left(e^{(\kappa +\gamma +\Delta )t}-1\right),\\ \langle A\left(p_{1}\right)A^{†}\left(p_{2}\right)\rangle & = & \langle A\left(p_{2}\right)A^{†}\left(p_{1}\right)\rangle =\frac{\left(n_{w}+1\right)}{\left(\kappa +\gamma \right)}\left(e^{(\kappa +\gamma )t}-1\right). \end{array}$$

Substituting these results for the correlation functions into Equation (A17), and taking the limit of t→∞, we obtain

(A25) $$\begin{array}{c} \lim_{t\to \infty }\langle b^{†}\left(t\right)b\left(t\right)\rangle =n_{b}+\frac{\kappa \left(n_{b}+1\right)g^{2}}{\left(\kappa +\gamma \right)\left(4\kappa \gamma -g^{2}\right)}+\frac{\kappa g^{2}}{\left(\kappa +\gamma \right)\left(4\kappa \gamma -g^{2}\right)}n_{w}. \end{array}$$

Writing $n_{w}$ in terms of $n=(n_{1}+n_{2})/2$, $\delta n=(n_{1}-n_{2})/2$, and $\tan\theta =g_{2}/g_{1}$, we obtain the expression for the population of the mode *b* given in Equation (10).
